# Associations of early life and childhood risk factors with obstructive sleep apnoea in middle‐age

**DOI:** 10.1111/resp.14592

**Published:** 2023-09-21

**Authors:** Chamara V. Senaratna, Adrian Lowe, E. Haydn Walters, Michael J. Abramson, Dinh Bui, Caroline Lodge, Bircan Erbas, John Burgess, Jennifer L. Perret, Garun S. Hamilton, Shyamali C. Dharmage

**Affiliations:** ^1^ Allergy & Lung Health Unit, Melbourne School of Population & Global Health The University of Melbourne Carlton Victoria Australia; ^2^ Faculty of Medical Sciences University of Sri Jayewardenepura Nugegoda Sri Lanka; ^3^ Non‐Communicable Diseases Research Centre University of Sri Jayewardenepura Nugegoda Sri Lanka; ^4^ Murdoch Children's Research Institute Melbourne Victoria Australia; ^5^ School of Medicine and Menzies Institute The University of Tasmania Hobart Tasmania Australia; ^6^ School of Public Health & Preventive Medicine Monash University Melbourne Victoria Australia; ^7^ School of Psychology and Public Health La Trobe University Melbourne Victoria Australia; ^8^ Violet Vines Marshman Centre for Rural Health Research La Trobe University Bendigo Victoria Australia; ^9^ Institute for Breathing and Sleep (IBAS) Heidelberg Victoria Australia; ^10^ Department of Lung, Sleep, Allergy and Immunology Monash Health Clayton Victoria Australia; ^11^ School of Clinical Sciences Monash University Clayton Victoria Australia

**Keywords:** adult, child, early‐life, obstructive sleep apnoea, risk factor, sleep‐disordered breathing

## Abstract

**Background and Objective:**

Early‐life risk factors for obstructive sleep apnoea (OSA) are poorly described, yet this knowledge may be critical to inform preventive strategies. We conducted the first study to investigate the association between early‐life risk factors and OSA in middle‐aged adults.

**Methods:**

Data were from population‐based Tasmanian Longitudinal Health Study cohort (*n* = 3550) followed from 1st to 6th decades of life. Potentially relevant childhood exposures were available from a parent‐completed survey at age 7‐years, along with previously characterized risk factor profiles. Information on the primary outcome, probable OSA (based on a STOP‐Bang questionnaire cut‐off ≥5), were collected when participants were 53 years old. Associations were examined using logistic regression adjusting for potential confounders. Analyses were repeated using the Berlin questionnaire.

**Results:**

Maternal asthma (OR = 1.5; 95% CI 1.1–2.0), maternal smoking (OR = 1.2; 1.05, 1.5), childhood pleurisy/pneumonia (OR = 1.3; 1.04, 1.7) and frequent bronchitis (OR = 1.2; 1.01, 1.5) were associated with probable OSA. The risk‐factor profiles of ‘parental smoking’ and ‘frequent asthma and bronchitis’ were also associated with probable OSA (OR = 1.3; 1.01, 1.6 and OR = 1.3; 1.01–1.9, respectively). Similar associations were found for Berlin questionnaire‐defined OSA.

**Conclusions:**

We found novel temporal associations of maternal asthma, parental smoking and frequent lower respiratory tract infections before the age of 7 years with adult OSA. While determination of their pathophysiological and any causal pathways require further research, these may be useful to flag the risk of OSA within clinical practice and create awareness and vigilance among at‐risk groups.

## Abstract 2

This study provides the first known evidence for individual and profiled early‐life and childhood risk factors for OSA in adults. It shows that early exposures to smoking and lower respiratory tract infections could be risks for adult OSA, which may stimulate future research and help flag future risk of OSA.

**See related** editorial

## INTRODUCTION

Obstructive sleep apnoea (OSA) is highly prevalent^1^ affecting a billion adults worldwide^2^ but remains underdiagnosed and under‐recognized^1^, ^2^, ^3^ with over half of those with OSA being asymptomatic or not recognizing symptoms.^4^ This high burden of undiagnosed OSA is concerning given its links with chronic cardiovascular disease,^5^ stroke,^5^ diabetes mellitus,^5^ depression,^6^ chronic obstructive pulmonary disease (COPD),^7^ asthma,^8^ cancer^9^ and mortality.^9^ Early diagnosis and treatment and primary preventive interventions could provide health and economic benefits to individuals and communities^10^ but are constrained by limited information available on longitudinal predictors of OSA.

To date, the known risk factors for OSA in adults include obesity (BMI >30 kg/m^2^),^5^, ^11^ central body fat distribution,^11^, ^12^ large neck circumference,^11^ male sex,^5^ older age,^5^ variant anatomy that causes crowding of the oropharyngeal and/or velopharyngeal region and collapsible and/or narrow upper airways,^5^ and genetic susceptibility as evident by familial inheritance^13^ of OSA and influence of ethnicity.^14^ Some adult OSA may have origins in the childhood.^15^ Anatomical risk factors for paediatric OSA could remain into adulthood even after therapeutic interventions.^16^, ^17^ Other childhood risk factors for adult OSA, however, has not yet been investigated and any study of perinatal and childhood risk factors have been performed only in relation to OSA in children.^18^, ^19^, ^20^

Investigation of the association of childhood risk factors with OSA in adults is ideally performed using prospective data available from childhood to adult life, but the paucity of such data precludes meaningful conclusions about such associations. This could be important knowledge needed to predict, screen for and diagnose OSA early in adults to provide them with beneficial interventions. Such knowledge could also trigger further research into the lifelong evolution and relevant pathophysiology of OSA. We aimed to bridge this gap in knowledge by assessing the associations of childhood health risk factors and risk factor profiles with OSA in middle‐age, which could provide new insights into delineating causal mechanisms and pathophysiology of OSA in adults.

## METHODS

### Study design and participants

We used data from the Tasmanian Longitudinal Health Study (TAHS),^21^ the largest and longest‐running respiratory health cohort study in the world. In 1968, TAHS recruited all children born in 1961 (aged 7‐years) and attending schools in Tasmania (probands; *n* = 8583) comprising 99% of the target population. They were followed up periodically, at least once a decade. Setting‐up of TAHS and subsequent follow‐ups have been described previously.^22^, ^23^ All surviving and contactable probands (*n* = 6128) were invited to participate in a survey and a clinical assessment at the age of 51–54 years (between 2012 and 2016) and 3609 (42% of the original cohort) responded.

### Data collection

#### Parental and early‐life risk factors

The potential risk factors for OSA that we investigated included currently known risk factors for childhood OSA^24^, ^25^ and general respiratory risk factors that we previously identified.^21^ Of these, the information on maternal and paternal asthma, maternal and paternal smoking, mode of feeding during the first 3 months or life (breast or bottle fed), doctor‐diagnosed pneumonia or pleurisy, food or drug allergies, hives, eczema, attacks of hay‐fever, frequency of bronchitis, frequency of asthma, history of tonsillectomy and bodyweight at age 7 years was gathered from the survey questions completed by the parents in 1968. Information on preterm (prematurity), birthweight and small for gestational age were extracted from hospital records. Late preterm was defined as born between 34 and 36 weeks + 6 days and moderate or very preterm were born before 34 weeks. Small for gestational age was birthweight <10th percentile for the duration of gestation. Normal birthweight was 2.5 to <4.0 kg. Childhood weight categories were defined using age‐ sex‐specific reference points.^26^, ^27^ Details of the survey questions that were used and definitions of variables are given in Appendix S1 in the Supporting Information.

#### Risk factor profiles

We previously considered 14 early life variables for a latent class analysis (LCA) and generated six risk profiles for general respiratory conditions.^21^ These were: (1) unexposed or least exposed to risk factors (the reference group), (2) parental smoking, (3) allergy, (4) infrequent asthma and bronchitis, (5) frequent asthma and bronchitis and (6) frequent asthma, bronchitis and allergy. Further details of the LCA and risk‐factor profiles are given in Appendix S2 in the Supporting Information.

#### Probable OSA

During the 53‐year follow‐up, the probands completed a self‐administered survey that included the STOP‐Bang^28^ (*S*noring, *T*iredness, *O*bserved apnoea, high blood *P*ressure, *B*MI, *A*ge, *N*eck circumference and *G*ender) and Berlin^29^ questionnaires (details in Appendix S3 in the Supporting Information) that detect probable OSA. These two questionnaires have standard cut‐off scores (≥3 for STOP‐Bang^28^ and ≥2 for Berlin^29^) but we found in our recent validation study in the same cohort^30^ that ≥5 was the optimum cut‐off score for STOP‐Bang, which was used in this analysis. The standard cut‐off score of ≥2 remained the optimal cut‐off score for the Berlin questionnaire and was used in this analysis.

### Statistical analysis

We reported descriptive data as numbers and percentages or means and SDs. Any differences in the relevant characteristics between those who did or did not respond to the OSA‐related questionnaires in the cohort were reported using the chi‐squared test. Logistic regression models were used to examine the associations of individual childhood risk factors and risk factor profiles with probable OSA. In these analyses, probable OSA was primarily reported using STOP‐Bang questionnaire, but all models were also refitted using Berlin questionnaire as a sensitivity analysis and reported in Tables S2 and S3 in the Supporting Information. Minimum sets of confounders developed using a directed acyclic graph (Figure S1 in the Supporting Information) were used in modelling. The results were reported as odds ratios (ORs) with 95% CIs. For risk factors and risk factor profiles that showed statistically significant associations with OSA, we performed relevant mediation analyses.^31^ The Stata/SE 17.0 software^32^ was used for statistical analysis.

## RESULTS

At the time of baseline data collection, the mean age ± SD of the probands was 6.5 ± 0.3 years (range 6.0–7.0 years). Other baseline characteristics are given in Table 1 and Table S1 in the Supporting Information. Of those who were invited to participate in the sixth decade follow‐up (*n* = 6128), a total of 3609 (59%) responded to survey questions. Information on childhood risk factors was available for 98% of the participants who responded to the STOP‐Bang questionnaire. The mean age ± SD at follow‐up was 53.0 ± 1.0 (range 50.8–55.6 years) and 48.9% were male. In the TAHS cohort (*n* = 8583), those who had OSA‐related information (i.e., those who participated in the sixth decade follow‐up) were more likely to be born preterm, have been bottle‐fed in the first 3 months of life, had never experienced hives and were less exposed to parental smoking, but more likely to have childhood eczema, hay‐fever, frequent bronchitis and history of tonsillectomy during childhood (Table S1 in the Supporting Information). Of the participants of sixth decade follow‐up, those who had OSA‐risk were more likely to have a history of maternal asthma, maternal smoking, frequent asthma by age 7 years and a higher frequency of pleurisy or pneumonia and frequent bronchitis compared with those who did not have an OSA‐risk, but less likely to have history of being solely fed on breast milk (Table 1).

**TABLE 1:** Distribution of childhood factors in those with or without probable OSA^a^ at 53 years of age.

		Those with probable OSA^a^ at age 53 years	Those without probable OSA^a^ at age 53 years	*p*
		N/n^b^ (%)	N/n^b^ (%)	
Parental factors				
Maternal asthma		71/508 (14.0)	286/2921 (9.8)	0.004
Maternal smoking (daily)		194/508 (38.2)	985/2921 (33.7)	0.050
Paternal asthma		48 /497 (9.7)	328/2872 (11.4)	0.249
Paternal smoking (daily)		297/494 (60.1)	1658/2868 (57.8)	0.336
Gestational, perinatal, feeding and weight‐related factors				
Preterm	Late preterm (34–36.9 weeks)	65/ 665 (9.6)	145/1239 (11.75)	0.076
	Moderate to very preterm (<34 weeks)	27/ 665 (4.1)	31/1239 (2.5)	
Birthweight	Low (<2.5 kg)	24/292 (8.2)	1513/1822 (83.0)	0.288
	High (>4.0 kg)	36/292 (12.3)	195/1822 (10.7)	
Small for gestational age		42/223 (18.8)	241/1426 (16.9)	0.476
Mode of feeding in first 3‐months	Breast milk only	211/522 (40.4)	1316/3014 (43.7)	0.005
	Bottle only	161/522 (30.8)	728/3014 (24.2)	
	Breast and bottle	150/522 (28.7)	970/3014 (32.2)	
Weight at age of 7 years	Underweight	38/1262 (3.0)	83/2162 (3.8)	0.189
	Overweight or obese	155/1262 (12.3)	224/2162 (10.4)	
Allergic conditions				
Food/drug allergy		40/421 (7.6)	224/3014 (7.4)	0.844
Hives (once or twice a year)		96/515 (18.6)	583/2981 (19.6)	0.627
Eczema		75.522 (14.4)	428/3001 (14.%)	0.949
Hay‐fever attacks		72/521 (13.8)	414/2997 (13.8)	0.997
Asthma frequency	Infrequent (<once in 3 months during past 2 years)	44/524 (8.4)	247/3024 (8.2)	
	Frequent (≥once in 3 months during past 2 years)	68/524 (13.0)	258/3024 (8.5)	0.004
Childhood infections and related factors				
Pleurisy/pneumonia	Once or twice	87/520 (16.7)	392/2999 (13.1)	0.025
Bronchitis frequency	Infrequent (<once in 3 months during past 2 years)	117/524 (22.3)	777/3024 (25.7)	0.033
	Frequent (≥once in 3 months during past 2 years)	148/524 (28.2)	704/3024 (23.3)	
History of tonsillectomy		211/1305 (16.2)	370/2239 (16.5)	0.782

Abbreviation: OSA, obstructive sleep apnoea.

^a^ Probable OSA = STOP‐Bang score ≥5.

^b^ Only those with childhood data were included.

The prevalence of probable OSA was 14.7% when defined using the STOP‐Bang questionnaire. Factors associated with probable OSA were maternal asthma (OR 1.5; 95% CI 1.1, 2.0), maternal smoking (OR 1.2; 1.05, 1.5), childhood pleurisy/pneumonia (OR 1.3; 1.04, 1.7) and frequent childhood bronchitis (OR 1.2; 1.01, 1.5; Table 2). The risk factor profiles of parental smoking (OR 1.3; 95% CI 1.01, 1.6) and frequent asthma and bronchitis in childhood (OR 1.3; 1.01–1.9) also were associated with a probable OSA (Table 3). These associations were confirmed when Berlin questionnaire was used in place of STOP‐Bang (Tables S2 and S3 in the Supporting Information).

**TABLE 2:** Association between childhood risk factors and probable OSA^a^ in middle‐age.

		Probable OSA^a^	
		Unadjusted models	Adjusted models
		OR (95% CI); *p*	OR (95% CI); *p*
Parental factors			
Maternal asthma		1.5 (1.1, 2.0); 0.005	1.5 (1.1, 2.0); 0.005^b^
Maternal smoking		1.2 (1.0, 1.5); 0.051	1.2 (1.05, 1.5); 0.044^c^
Paternal asthma		0.8 (0.6, 1.1); 0.250	0.8 (0.6, 1.1); 0.250^b^
Paternal smoking		1.1 (0.9, 1.3); 0.336	1.1 (0.9, 1.3); 0.351^d^
Gestational, perinatal, feeding and weight‐related factors			
Preterm^e^	Late preterm	0.8 (0.5, 1.2); 0.297	0.8 (0.5, 1.2); 0.291^f^
	Moderate or very preterm	1.2 (0.6, 2.4); 0.679	1.3 (0.6, 2.8); 0.454^f^
Birthweight	Low birthweight (<2.5 kg)	1.3 (0.9, 2.2); 0.178	1.0 (0.5, 2.0);0.869^g^
	High birthweight (≥4 kg)	1.2 (0.8, 1.8); 0.340	1.2 (0.8, 2.0); 0.327^g^
Small for gestational age		1.1 (0.8, 1.6); 0.477	1.1 (0.8, 1.6); 0.636^f^
Mode of feeding in first 3‐months	Bottle only	1.4 (1.1, 1.7); 0.005	1.0 (0.6, 1.4); 0.793^h^
	Breast and bottle	0.7 (0.3, 1.3); 0.173	0.8 (0.6, 1.1); 0.114^h^
BMI at age 7 years^i^	Underweight	0.7 (0.4, 1.3); 0.318	1.1 (0.5, 2.4); 0.896^j^
	Overweight	0.8 (0.6, 1.2); 0.283	0.8 (0.5, 1.4); 0.471^j^
	Obese	1.4 (0.7, 2.6); 0.329	1.2 (0.4, 3.5); 0.766^j^
Allergic conditions			
Food/drug allergy		1.0 (0.7, 1.5); 0.844	1.0 (0.7, 1.5); 0.844^b^
Hives		0.9 (0.7, 1.2); 0.627	0.9 (0.7, 1.2); 0.627^b^
Eczema		1.0 (0.8, 1.3); 0.949	1.0 (0.8, 1.3); 0.949^b^
Hay‐fever attacks		1.0 (0.8, 1.3); 0.997	1.0 (0.8, 1.3); 0.997^b^
Asthma frequency	Infrequent (<once in 3 months during past 2 years)	1.1 (0.8, 1.5); 0.620	0.8 (0.6, 1.2); 0.431^k^
	Frequent (≥once in 3 months during past 2 years)	1.6 (1.2, 2.1); 0.001	1.1 (0.8, 1.6); 0.434^k^
Childhood infections and related factors			
Pleurisy/pneumonia	Once or twice	1.3 (1.03, 1.7); 0.025	1.3 (1.04, 1.7); 0.023^l^
Bronchitis frequency	Infrequent (<once in 3 months during past 2 years)	0.9 (0.7, 1.1); 0.364	0.8 (0.6, 1.1); 0.133^l^
	Frequent (≥once in months during past 2 years)	1.2 (1.01, 1.6); 0.046	1.2 (1.01, 1.5); 0.049^l^
History of tonsillectomy		1.0 (0.8, 1.3); 0.768	1.0 (0.8, 1.3); 0.768^b^

Abbreviation: OSA, obstructive sleep apnoea.

^a^ Probable OSA = STOP‐Bang score ≥5.

^b^ No adjustment needed as per the causal model (Figure S1 in the Supporting Information).

^c^ Adjusted for maternal asthma.

^d^ Adjusted for paternal asthma.

^e^ Results did not materially change when analysed as a dichotomised variable (full‐term vs. preterm).

^f^ Adjusted for maternal smoking and paternal smoking.

^g^ Adjusted for maternal smoking, paternal smoking, prematurity and small for gestational age.

^h^ Adjusted for prematurity, small for gestational age and birthweight.

^i^ Results did not materially change when analysed as a dichotomised variable (normal weight vs. overweight or obese).

^j^ Adjusted for prematurity, small for gestational age, birthweight and breast/bottle feeding.

^k^ Adjusted for maternal asthma, and paternal asthma, maternal smoking, paternal smoking, gender, breast/bottle feeding and BMI at age 7 years.

^l^ Adjusted for childhood BMI.

**TABLE 3:** Association between childhood risk factor‐profiles and probable OSA^a^.

Risk‐factor profiles	Probable OSA^a^	
	Unadjusted models	Adjusted models
	OR (95% CI); *p*	OR (95% CI); *p*
Unexposed or least exposed (reference group)	1.0	1.0
Parental smoking	1.2 (1.0, 1.6); 0.082	1.3 (1.01, 1.6); 0.048^b^
Allergy	1.2 (0.7, 1.8); 0.990	1.2 (0.7, 1.8); 0.990^c^
Infrequent asthma and bronchitis	1.0 (0.7, 1.4); 0.943	0.8 (0.5, 1.3); 0.306^d^
Frequent asthma and bronchitis	1.6 (1.2, 2.2); 0.002	1.3 (1.01, 1.9); 0.049^d^
Frequent asthma, bronchitis and allergy	1.4 (0.8, 2.4); 0.187	0.9 (0.5, 1.7); 0.775^d^

Abbreviation: OSA, obstructive sleep apnoea.

^a^ Probable OSA = STOP‐Bang score ≥5.

^b^ Adjusted for maternal asthma and paternal asthma as per the causal model (see Figure S1 in the Supporting Information).

^c^ No adjustment needed as per the causal model.

^d^ Adjusted for maternal smoking, paternal smoking, maternal asthma, paternal asthma, breast/bottle feeding, childhood BMI and gender.

We investigated if the associations of maternal asthma, smoking exposure and childhood lower respiratory infections with probable OSA were mediated through adult asthma and adult COPD as both these are associated with OSA^33^, ^34^ as well as with the childhood risk factors.^35^, ^36^, ^37^ We found no significant mediation for any of the variables (data not shown).

## DISCUSSION

This is the first study to assess the role of prenatal and early childhood factors on OSA in middle‐age. We found that maternal asthma and smoking, lower respiratory tract infections and the risk‐factor profiles of parental smoking and frequent asthma and bronchitis in childhood were associated with a probable OSA in middle‐age.

Although these associations are longitudinal, it is difficult to determine if they are causal or mere predictors of OSA given the current understanding of the pathophysiology of OSA. There are four widely accepted main pathophysiological pathways:^38^ an anatomically or functionally collapsible upper airway; poor upper airway dilator muscle responsiveness; high loop‐gain of the respiratory system causing respiratory control instability; and low respiratory arousal threshold. Nevertheless, it is difficult to directly link any of the risk factors that we detected to these pathophysiological pathways. For example, this current knowledge makes it difficult to theorize how maternal asthma would cause OSA in adult offspring. However, as OSA symptoms might be misconstrued as nocturnal asthma,^39^ at least some of the maternal asthma could be maternal OSA and may indicate a familial inheritance of OSA as previously shown.^13^ Furthermore, maternal asthma could also suggest abnormal airways, a trait the offspring could inherit and be associated with adult OSA. However, the pathophysiology of this association should be explored in future research and the studies of OSA epidemiology must explore maternal asthma as an alternative pathway to OSA. Similarly, the pathophysiology of the association between early exposure to smoking and subsequent OSA remains to be sufficiently characterized. Yet, given that passive smoking has been linked to childhood OSA in at least some studies,^24^, ^40^ it is possible that this association could, at least partially, be due to childhood OSA persisting to the adult life.

Although obesity in adults was associated with OSA,^41^ BMI at the age of 7 years was not associated with OSA in adults. We have previously shown that the BMI from childhood to middle age takes five different trajectories,^42^ and if the effect of BMI on OSA was immediate rather than long‐lasting as previously shown,^43^ this would explain the lack of association between childhood BMI and OSA in adults. Similar to childhood obesity, other risk factors for childhood OSA^20^ such as prematurity and feeding mode were not associated with OSA in adults suggesting different pathophysiological pathways for OSA in children and adults.

Recurrent respiratory tract infections are associated with childhood OSA,^44^ and so it is likely that at least some of these adults with OSA had childhood OSA which continued into adult life. Childhood lower respiratory tract infections are associated with restrictive and obstructive lung diseases^45^ which are also associated with OSA.^33^, ^46^ However, our mediation analysis showed no significant mediation of these observed associations via asthma or COPD in adult life. In adults, an association of chronic bronchitis with OSA^47^ and OSA symptoms^48^ had been shown. However, we demonstrated that childhood bronchitis is not associated with chronic bronchitis in adults^49^ so our finding of association of bronchitis in children with OSA in adults is novel. Frequent lower respiratory tract infections including bronchitis also retard the growth of the respiratory system,^50^ and any resulting anatomical or functional changes that narrow the airways and/or make them collapsible would likely increase the risk of OSA.^38^ On the other hand, the likelihood of an unobserved latent factor driving both respiratory tract infections during childhood and OSA in the adult life cannot be excluded.

Our study has both strengths and limitations. The main advantage in our prospective population‐based design is that it has eliminated potential recall bias and ensured wide generalizability. By using the Berlin questionnaire in sensitivity analyses, we have enabled consistent and triangulated observations which strengthen the validity of our findings. These two questionnaires have different sensitivity and specificity levels,^30^ yet showed similar results for the associations. However, despite these data coming from a cohort study, we cannot attribute causal inferences to our findings as OSA was detected from cross‐sectional data rather than true incident data due to lack of data on childhood OSA. Therefore, the detected associations would best serve to generate hypotheses, which must be tested in further studies.

The main limitation of our study was the reliance on STOP‐Bang ≥5 to define OSA instead of sleep studies. STOP‐Bang ≥5 has high specificity but less sensitivity^30^ and therefore, we may have undercounted OSA. Fortunately, any such misclassification is likely to be non‐differential and if so, would have underestimated the observed associations and may also be a reason why some risk‐factors were detected with marginal statistical significance. It could also mean some true associations between potential risk factors and OSA went undetected in our study. Sleep studies may detect stronger associations but would be logistically difficult with the numbers required for epidemiological studies. Any studies with polysomnography would likely have much smaller numbers to analyse and so lack statistical power. Missing childhood data for some exposure variables in our study may also have led to reduced statistical power. With multiple modelling using individual variables and likely risk factor profiles, the probability of us detecting a spurious association increased and therefore, these results must be interpreted with caution.

Despite these limitations and the inability to draw causal inferences, this first known study to investigate the role of both individual and profiles of childhood risk factors reinforced the suggestions of childhood origins of adult OSA^15^, ^16^, ^17^ and provided new insights into modifiable early life risk factors for OSA, which may stimulate future research. There were strong signals that early exposures to smoking and lower respiratory tract infections could be risks for adult OSA, which could be used in general practice as a part of the routine clinical check‐ups to identify those who may be at risk of developing OSA. This knowledge could also be used in a larger scale in public health education to create population awareness, enabling those at risk to be vigilant of OSA which is likely to help detect any OSA early.

In conclusion, maternal asthma, parental smoking and frequent lower respiratory tract infections in childhood were associated with a probable OSA in middle‐age. These findings are a likely stepping‐stone for further research on early life risk factors for OSA in adults and provide a platform to investigate plausible pathophysiological mechanisms.

## AUTHOR CONTRIBUTIONS

**Chamara V. Senaratna:** Conceptualization (equal); data curation (supporting); formal analysis (lead); investigation (supporting); methodology (equal); visualization (lead); writing – original draft (lead); writing – review and editing (lead). **Adrian Lowe:** Funding acquisition (supporting); investigation (supporting); project administration (supporting); supervision (supporting); writing – review and editing (supporting). **E. Haydn Walters:** Funding acquisition (supporting); investigation (supporting); writing – review and editing (supporting). **Michael J. Abramson:** Funding acquisition (supporting); investigation (supporting); writing – review and editing (supporting). **Dinh Bui:** Formal analysis (supporting); investigation (supporting); methodology (equal); writing – review and editing (supporting). **Caroline Lodge:** Funding acquisition (supporting); investigation (supporting); project administration (supporting); writing – review and editing (supporting). **Bircan Erbas:** Funding acquisition (supporting); investigation (supporting); writing – review and editing (supporting). **John Burgess:** Investigation (supporting); writing – review and editing (supporting). **Jennifer L. Perret:** Funding acquisition (supporting); investigation (supporting); project administration (supporting); supervision (supporting); writing – review and editing (supporting). **Garun S. Hamilton:** Conceptualization (equal); funding acquisition (supporting); investigation (supporting); methodology (equal); supervision (equal); validation (equal); writing – review and editing (supporting). **Shyamali C. Dharmage:** Conceptualization (equal); data curation (lead); funding acquisition (lead); investigation (lead); methodology (lead); project administration (lead); resources (lead); supervision (lead); validation (lead); writing – original draft (supporting); writing – review and editing (supporting).

## CONFLICT OF INTEREST STATEMENT

Michael Abramson and Bircan Erbas are Statistical Review Board members of *Respirology* and co‐authors of this article. They were excluded from all editorial decision‐making related to the acceptance of this article for publication.

Adrian Lowe, Michael J. Abramson and Jennifer L. Perret have received Australian National Health and Medical Research Council grants unrelated to the current project. Michael J. Abramson has also received grants from Pfizer, Boehringer Ingelheim and personal fees and other from Sanofi and GSK. Jennifer L. Perret has received grants from Boehringer Ingelheim. Adrian Lowe has received non‐financial support from Primus Pharmaceuticals for unrelated research. Garun S. Hamilton has received research support and equipment unrelated to the current work from ResMed, Phillips Respironics and Air Liquide Healthcare. Other authors have no conflicts of interests to declare.

## HUMAN ETHICS APPROVAL DECLARATION

This study was conducted in accordance with the amended Declaration of Helsinki and was approved by the Human Research Ethics Committee of the University of Tasmania (approval number, H0012710). Participants provided written informed consent.

## Supporting information


**Data S1.** Supporting Information.

## Data Availability

Any additional information regarding data in this manuscript are available from the corresponding author.
