# Supplementary material for: Associations of early life and childhood risk factors with obstructive sleep apnoea in middle‐age
Source: Respirology. 2023 Sep 21;29(1):63–70. doi: 10.1111/resp.14592 (PMC10952926; doi:10.1111/resp.14592)
Supplement: Supplementary file 1 — Data S1. Supporting Information. [file RESP-29-63-s001.docx]

**Associations of early life and childhood risk factors with obstructive sleep apnoea in middle-age**

**Supporting Information**

**Appendix S1**

***Childhood exposure variables:***

Maternal and paternal smoking were defined by parents’ responses to the question “Do you smoke every day (or six days out of seven)?”.

Maternal and paternal asthma were defined by parents’ responses to the question “Have you ever had asthma or attacks of wheezing like asthma?”.

**Preterm, small for gestational age, and birthweight were defined using birth related information obtained from the parents and hospital records.** **Late preterm was defined as being born between 34+0⁄7 weeks and 36+6⁄7 and moderate or very preterm was defined as being born before 34 weeks. Small for gestational age was when the birthweight was <10^th^ percentile for a particular duration of gestation. Birthweight was classified as low if <2.5kg, normal if 2.5-<4.0kg and high if ≥4.0kg.**

Mode of feeding during the first three months of life was defined by the question “How was he/she fed in the first three months of life?” and included three categories: breast only, bottle only, and both breast and bottle.

Childhood weight status (overweight, obesity, and underweight) was defined using the age and sex-specific cut points from the reference tables of Cole et al. ^1,2^

Childhood food allergy was defined by the question “Have you been told by a doctor that he/she is allergic to any foods or medicines?”

Childhood hives (urticaria) was defined by the question “Does he/she get hives?”

Childhood eczema was defined by an affirmative response to either of the two questions “Does he/she have infantile (baby) eczema?” and “Has he/she ever had eczema in the creases of elbows, wrists, or knees?”.

Childhood hay fever was defined by the question “Does he/she get attacks of hay fever?”.

Childhood asthma included three categories: never, infrequent asthma and frequent asthma. This variable was defined by two questions: “Has he/she at any time in his/her life suffered from attacks of asthma or of wheezy breathing?” and “On the average, how often do these attacks tend to occur over the last two years or so?”. Frequent asthma was defined as one or more attacks every three months during the past two years. Infrequent asthma was defined as having the frequency of attacks less than every three months during the past two years.

Childhood lung infections were defined by the question “Have you ever been told by a doctor that he/she had pneumonia or pleurisy?”

Childhood bronchitis included three categories: never, infrequent bronchitis and frequent bronchitis. This variable was defined by two questions: “Has he/she at any time in his/her life suffered from attacks of bronchitis or attacks of bronchitis with sputum in the chest?” and “On the average, how often do these attacks tend to occur over the last two years or so?”. Frequent bronchitis was defined as one or more attacks every three month during the past two years.

History of tonsillectomy was defined using a question “Have your child’s tonsils been removed surgically?”.

**Appendix S2**

***LCA model selection for risk factors profiles:^3^***

All questions about respiratory illness and risk factors collected at 7 years were extracted for this analysis. Thirteen variables were initially included to identify risk factor profiles using latent class analysis (LCA). They included childhood asthma (included in three categories: never, infrequent and frequent), bronchitis (also in three categories), eczema, hay fever, food allergy, “hives”, lung infections, breast feeding, weight status, maternal smoking, paternal smoking, maternal asthma and paternal asthma. Breast feeding and childhood weight status were later excluded from the final LCA model because they were found not to differentiate between classes (i.e. classes had similar probability of having these two variables).

***Childhood risk factor profiles identified by LCA:***

Six risk profiles (latent classes) were labelled based on the probability of risk factors in each profile relatively to the population.

1. The profile characterized by the lowest probability of any risk factors was labelled as “unexposed or least exposed” (49.0%)

2. The profile characterized by the highest probability of both maternal smoking and paternal smoking but probability of other risk factors being similar to the “unexposed or least exposed” profile was labelled as “parental smoking” (21.5%).

3. The profile characterized by medium probability of eczema, food allergy, hay-fever and hives, low probability of bronchitis, and other risk factors being similar to the “unexposed or least exposed” profile was labelled as “allergy” (10.0%).

4. The profile characterized dominantly by medium/high probability of both frequent asthma attacks and frequent bronchitis attacks but not other risk factors was labelled as “frequent asthma, bronchitis” (8.7%).

5. The profile characterized dominantly by medium/high probability of both infrequent asthma attacks and infrequent bronchitis attacks but not other risk factors was labelled as “infrequent asthma, bronchitis” (8.3%).

6. The profile characterized by the highest probability of frequent asthma attacks, frequent bronchitis attacks, eczema, food allergy and hay-fever was labelled as “frequent asthma, bronchitis and allergy” (2.6%).

**Appendix S3**

**OSA-screening questionnaires**

STOP-Bang questionnaire has eight-items that questions on snoring, tiredness, cessation of breathing in sleep, presence of hypertension, age, and gender and information on BMI and neck circumference. Each question or measurement is equally scored. A score of at least 3 out of the total of 8 suggests a high risk of OSA.^4^

Berlin questionnaire (BQ) has three categories comprised of information on height and weight and ten questions. These questions/information and categories are: snoring and cessation of breathing (category 1; five questions); symptoms of fatigue and excessive daytime sleepiness (Category 2; four questions); and body-mass index (BMI; derived from height and weight measurements) and hypertension (category 3; height, weight information and one question, respectively). One point each was given to the responses that indicated presence, higher intensity, or higher frequency of symptoms. No points were given to the responses that indicated absence, lower intensity, or lower frequency of symptoms. These points within a given category were then considered to determine if that category is positive or negative: Category 1 was considered positive if it had at least two points; Category 2 was considered positive if it had at least two points; and Category 3 was considered positive if respondents had hypertension (one point) or BMI >30 kg/m^2^. Presence of at least two positive categories would indicate high risk for OSA.^5^


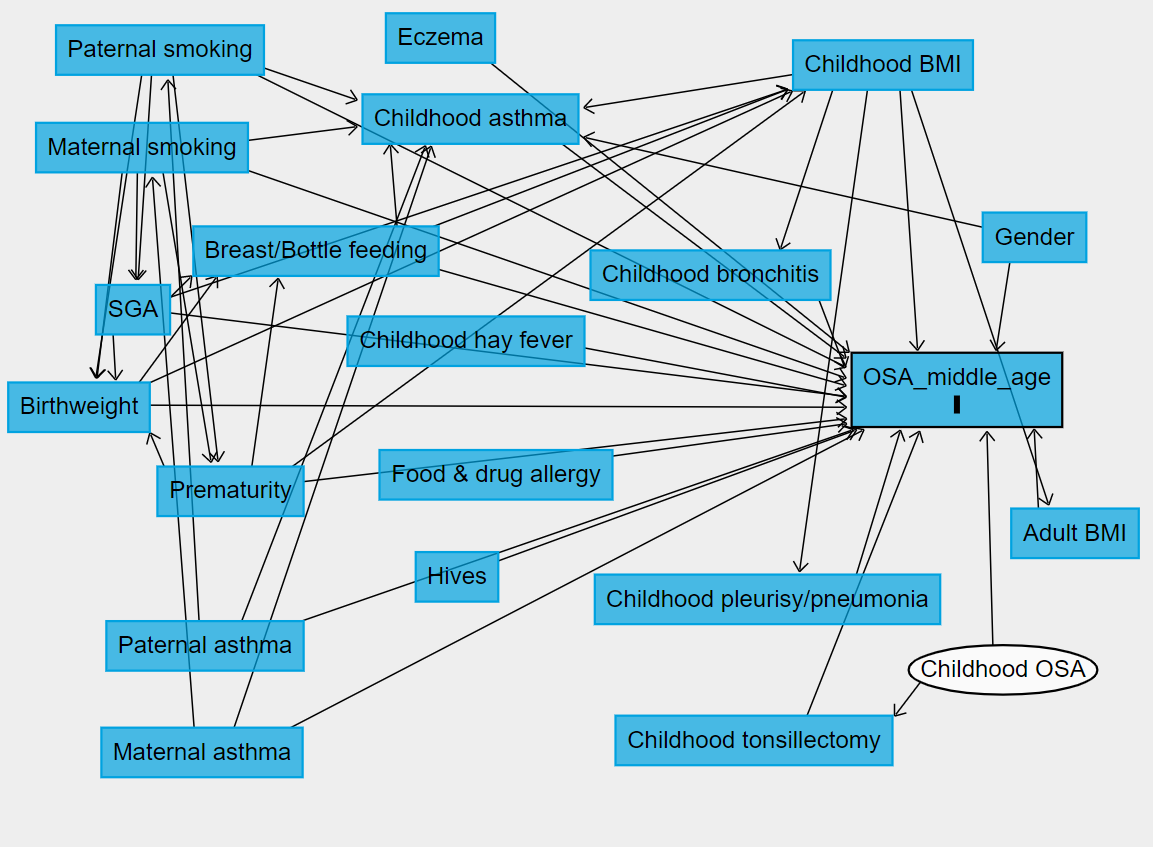


**Figure S1. Causal diagram for the association of childhood and early life risk factors with OSA in adulthood (SGA=small for gestational age; BMI=body-mass index; OSA=obstructive sleep apnoea).**

**Table S1. Distribution of childhood factors and risk profiles among those who have and not have OSA-related data**

|  |  | **Have OSA-related data (n=3,609)** | **Do not have OSA-related data (not followed up; n=4,974)** | ^†^**p** |
| --- | --- | --- | --- | --- |
|  |  | **N (%)** | **N (%)** |  |
| ***Parental factors*** |  |  |  |  |
| Maternal asthma | Has asthma | 357 (10.1) | 519 (10.8) | 0.136 |
| Maternal smoking | Smoking daily | 1179 (33.2) | 1824 (38.0) | **<0.001** |
| Paternal asthma | Has asthma | 376 (10.6) | 462 (9.6) | 0.324 |
| Paternal smoking | Smoking daily | 1955 (55.1) | 2811 (58.5) | **<0.001** |
| ***Gestational, natal, feeding and weight-related factors*** |  |  |  |  |
| Preterm | Late preterm | 209 (11.0) | 104 (6.3) | **<0.001** |
|  | Moderate or very preterm | 58 (3.0) | 40 (2.4) |  |
| Birthweight | Low (<2.5 kg) | 138 (2.5) | 118 (3.9) | 0.275 |
|  | High (>4.0 kg) | 231 (6.5) | 205 (4.3) |  |
| Small for gestational age |  | 283 (17.7) | 282 (19.2) | 0.136 |
| Mode of feeding in first 3-months | Breast only | 889 (25.4) | 1478 (30.8) | **<0.001** |
|  | Bottle only | 2647 (74.6) | 3266 (68.0) |  |
|  | Breast and bottle | 14 (0.4) | 58 (1.2) |  |
| Underweight/overweight during childhood | Underweight | 121 (3.4) | 159 (3.3) | 0.514 |
|  | Overweight or obese | 62 (1.8) | 68 (1.4) |  |
| ***Allergic conditions*** |  |  |  |  |
| Food/drug allergy | Has allergies | 264 (7.4) | 306 (6.4) | 0.067 |
| Hives | Once or twice a year | 679 (19.1) | 1005 (20.9) | **0.028** |
| Eczema | Had eczema | 503 (14.2) | 597 (12.4) | **0.024** |
| Hay-fever attacks | Gets attacks | 486 (13.7) | 575 (12.0) | **0.020** |
| Asthma frequency | Infrequent (<once in three months during past 2 years) | 291 (8.2) | 370 (7.7) | 0.425 |
|  | Frequent (≥once in three months during past 2 years) | 326 (9.2) | 412 (8.6) |  |
| ***Childhood infections and related factors*** |  |  |  |  |
| Pleurisy/pneumonia | Once or twice | 479 (13.5) | 698 (14.5) | 0.139 |
| Bronchitis frequency | Infrequent (<once in three months during past 2 years) | 894 (25.2) | 1196 (24.9) | **0.005** |
|  | Frequent (≥once in three months during past 2 years) | 852 (24.0) | 1018 (21.2) |  |
| History of tonsillectomy |  | 581 (16.4) | 707 (14.8) | **0.041** |

^†^p is for the chi-squared test (2x2 or nx2).

**Table S2. Association between childhood risk factors and probable OSA (defined using Berlin questionnaire)**

|  |  | **Berlin questionnaire-defined probable OSA** | |
| --- | --- | --- | --- |
|  |  | **Unadjusted models** | **Adjusted models** |
|  |  | **OR (95% CI); p** | **OR (95% CI); p** |
| ***Parental factors*** |  |  |  |
| Maternal asthma |  | 1.2 (1.0, 1.5); 0.059 | ^a^1.2 (1.0, 1.5); 0.059 |
| Maternal smoking | Smoking daily | 1.2 (1.03, 1.4); **0.017** | ^b^ 1.2 (1.03, 1.4); **0.017** |
| Paternal asthma |  | 1.1 (0.8, 1.3); 0.589 | ^a^1.1 (0.8, 1.3); 0.589 |
| Paternal smoking | Smoking daily | 1.2 (1.08, 1.4); **0.002** | ^c^ 1.2 (1.1, 1.4); **0.003** |
| ***Gestational, natal, feeding and weight-related factors*** |  |  |  |
| Preterm | Late preterm | 0.8 (0.6, 1.1); 0.204 | ^d^ 0.8 (0.6, 1.1); 0.195 |
|  | Moderate or very preterm | 1.6 (0.9, 2.7); 0.075 | ^d^ 1.9 (1.1, 3.4); **0.021** |
| Birthweight | Low birthweight (<2.5kg) | 1.4 (1.04, 2.1); **0.029** | ^e^ 1.6 (1.0, 2.6); 0.053 |
|  | High birthweight (>4kg) | 1.2 (0.9, 1.6); 0.124 | ^e^ 1.2 (0.8, 1.6); 0.344 |
| Small for gestational age |  | 1.0 (0.7, 1.3); 0.882 | ^d^ 0.9 (0.7, 1.2); 0.665 |
| Mode of feeding in first 3-months | Bottle only | 1.2 (1.04, 1.5); **0.017** | ^f^ 0.9 (0.7, 1.2); 0.528 |
|  | Breast and botte | 1.0 (0.9, 1.2); 0.886 | ^f^ 1.0 (0.8, 1.3); 0.813 |
| BMI at age 7 years | Underweight | 0.8 (0.5, 1.2); 0.249 | ^g^ 1.1 (0.6, 2.0); 0.793 |
|  | Overweight | 1.2 (1.0, 1.6); 0.076 | ^g^ 1.2 (0.9, 1.8); 0.218 |
|  | Obese | 1.0 (0.6, 1.7); 0.931 | ^g^ 0.9 (0.4, 2.1); 0.797 |
| ***Allergic conditions*** |  |  |  |
| Food/drug allergy |  | 1.2 (1.0, 1.6); 0.083 | ^a^1.2 (1.0, 1.6); 0.083 |
| Hives |  | 1.1 (0.9, 1.3); 0.404 | ^a^1.1 (0.9, 1.3); 0.404 |
| Eczema |  | 1.0 (0.8, 1.2); 0.775 | ^a^1.0 (0.8, 1.2); 0.775 |
| Hay-fever attacks |  | 1.0 (0.8, 1.2); 0.761 | ^a^1.0 (0.8, 1.2); 0.761 |
| Asthma frequency | Infrequent (<once in 3 months during past 2 years) | 1.0 (0.8, 1.3); 0.872 | ^h^ 0.9 (0.7, 1.2); 0.618 |
|  | Frequent (≥once in 3 months during past 2 years) | 1.3 (1.01, 1.6); **0.042** | ^h^ 1.2 (0.9, 1.5); 0.265 |
| ***Childhood infections*** |  |  |  |
| Pleurisy/pneumonia | Once or twice | 1.3 (1.03, 1.5); **0.023** | ^i^ 1.2 (1.02, 1.5); **0.030** |
| Bronchitis frequency | Infrequent (<once in months during past 2 years) | 1.0 (0.9, 1.2); 0.804 | ^i^ 1.0 (0.8, 1.2); 0.996 |
|  | Frequent (≥once in months during past 2 years) | 1.2 (1.05, 1.5); **0.011** | ^i^ 1.2 (1.02, 1.4); **0.027** |
| History of tonsillectomy |  | 1.0 (0.8, 1.2); 0.782 | ^a^1.0 (0.8, 1.2); 0.782 |

^a^ No adjustment needed as per the causal model (Figure S1); ^b^ Adjusted for maternal asthma; ^c^ Adjusted for paternal asthma; ^d^ Adjusted for maternal smoking and paternal smoking; ^e^ Adjusted for maternal smoking, paternal smoking, prematurity, and small for gestational age; ^f^ Adjusted for prematurity, small for gestational age, and birthweight;  ^g^ Adjusted for prematurity, small for gestational age, birthweight, and breast/bottle feeding; ^h^ Adjusted for maternal asthma, and paternal asthma, maternal smoking, paternal smoking, gender, breast/bottle feeding, and BMI at age 7 years; ^i^ Adjusted for childhood BMI; BMI=Body-mass index; OSA=Obstructive sleep apnoea

**Table S3. Association between childhood risk factor-profiles and probable OSA (defined using Berlin questionnaire)**

|  | **Berlin questionnaire-defined probable OSA** | |
| --- | --- | --- |
|  | **Unadjusted models** | **Adjusted models** |
| **Risk factor profiles** | **OR (95% CI); p** | **OR (95% CI); p** |
| Unexposed or least exposed (Reference group) | 1.0 | 1.0 |
| Parental smoking | 1.2 (1.04, 1.5); **0.016** | ^a^ 1.3 (1.06, 1.5); **0.011** |
| Allergy | 1.2 (0.8, 1.6); 0.366 | ^b^ 1.2 (0.8, 1.6); 0.366 |
| Infrequent asthma & bronchitis | 1.2 (0.9, 1.5); 0.222 | ^c^ 1.1 (0.8, 1.4); 0.587 |
| Frequent asthma & bronchitis | 1.4 (1.1, 1.8); **0.003** | ^c^ 1.4 (1.01, 1.8); **0.049** |
| Frequent asthma, bronchitis, & allergy | 1.4 (0.9, 2.1); 0.115 | ^c^ 1.2 (0.8, 1.9); 0.363 |

^a^ Adjusted for maternal asthma and paternal asthma as per the causal model (see Figure S1); ^b^ No adjustment needed as per the causal model; ^c^ Adjusted for maternal smoking, paternal smoking, maternal asthma, paternal asthma, breast/bottle feeding, childhood BMI, and gender; OSA=Obstructive sleep apnoea; BMI=Body-mass index

**References**

1. Cole TJ, Bellizzi MC, Flegal KM, Dietz WH. Establishing a standard definition for child overweight and obesity worldwide: international survey. BMJ. 2000;320(7244):1240.

2. Cole TJ, Flegal KM, Nicholls D, Jackson AA. Body mass index cut offs to define thinness in children and adolescents: international survey. BMJ : British Medical Journal. 2007;335(7612):194-.

3. Bui DS, Walters HE, Burgess JA, Perret JL, Bui MQ, Bowatte G, et al. Childhood respiratory risk factor profiles and middle-age lung function: a prospective cohort study from the first to sixth decade. Ann Am Thorac Soc. 2018;15(9):1057-66.

4. Chung F, Yegneswaran B, Liao P, Chung SA, Vairavanathan S, Islam S, et al. STOP questionnaire: a tool to screen patients for obstructive sleep apnea. Anesthesiology. 2008;108(5):812-21.

5. Netzer NC, Stoohs RA, Netzer CM, Clark K, Strohl KP. Using the Berlin Questionnaire to identify patients at risk for the sleep apnea syndrome. Ann Intern Med. 1999;131(7):485-91.
